# Erratum

**DOI:** 10.1093/ehjdh/ztab106

**Published:** 2021-12-29

Upon the original publication of the below listed articles, the Data Availability statement was inadvertently omitted. This erratum has been published to address the omission and note that the statements have been instated for the following papers:

Back to the vinyl age: a narrative report of a total computer blackout at a large university medical centre

Lorenz H. Lehmann and Benjamin Meder

*European Heart Journal - Digital Health*, Volume 2, Issue 1, March 2021, Pages 167–170, https://doi.org/10.1093/ehjdh/ztab006

Voice-based screening for SARS-CoV-2 exposure in cardiovascular clinics

Abhinav Sharma, Emily Oulousian, Jiayi Ni, Renato Lopes, Matthew Pellan Cheng, Julie Label, Filipe Henriques, Claudia Lighter, Nadia Giannetti, and Robert Avram

*European Heart Journal - Digital Health*, Volume 2, Issue 3, September 2021, Pages 521–527, https://doi.org/10.1093/ehjdh/ztab055

